# Stress granule phase transitions and neuronal fate in cerebral ischemia-reperfusion injury

**DOI:** 10.3389/fnmol.2026.1927505

**Published:** 2026-09-07

**Authors:** Xue Liao, Li Li, Xiaohong Zhang, Qin Chen, Chunyan Guo, Jinsong Luo, Tian Wang, Zuhong Wang

**Affiliations:** 1 Xinjin District Hospital of Traditional Chinese Medicine, Chengdu, China; 2 Yunnan University of Chinese Medicine, Kunming, China; 3 The Third Affiliated Hospital of Yunnan University of Chinese Medicine, Kunming, China; 4 Langzhong Hospital of Traditional Chinese Medicine, Langzhong, China

**Keywords:** cerebral ischemia-reperfusion injury, liquid-liquid phase separation, neuroprotection, proteostasis, stress granules

## Abstract

Cerebral ischemia-reperfusion injury (CIRI) drives neuronal death through secondary molecular events that persist after blood flow is restored. How neurons commit to survival or death before individual death programs engage remains unclear. Liquid-liquid phase separation and its stress granules (SGs) offer one regulatory platform. Under CIRI stress, SGs assemble around G3BP1 and concentrate stalled mRNP complexes with RNA-binding proteins such as TDP-43, FUS, and DDX3X. The physical state of the condensate sets its function. Liquid-state SGs are cytoprotective. In a mechanism so far demonstrated outside CIRI itself, they sequester executioner caspase-3 and caspase-7. They also upregulate GPX4 protein through a G3BP1-IGF2BP1-m6A hub that limits ferroptosis. They further reduce DDX3X availability for NLRP3 inflammasome assembly, an effect now supported by direct evidence in ischemic brain tissue. When injury exceeds what liquid condensates can buffer, oxidative modification drives an irreversible liquid-to-solid transition. Cytoplasmic mislocalization of TDP-43 after nuclear pore damage, progressive FUS aggregation under sustained oxidative stress, and chaperone depletion accelerate this shift. The result is proteostasis collapse through joint failure of the ubiquitin-proteasome system and selective autophagy. Rodent occlusion models place this bifurcation in early reperfusion, broadly within the first day, though estimates remain approximate and the human interval is undefined. Muscone and icariin may stabilize acute-phase condensates, while melatonin and HDAC6 inhibition may resolve subacute aggregates. The account moves from the biophysical basis of SG formation through the distinct ischemic and reperfusion phases of assembly. It then covers the bifurcating protective and pathological trajectories before turning to therapeutic strategies and their translational limitations. This review argues that the SG checkpoint is an underappreciated node in CIRI. Progress requires defining its time window in human neurons, resolving SG behavior across the neurovascular unit, and validating topology-targeting approaches in primates.

## 1 Introduction

Cerebral ischemia-reperfusion injury (CIRI) is one of the most consequential and mechanistically complex problems in clinical neuroscience (Zhang et al., 2024). Restoring blood flow after ischemic stroke is the prerequisite for tissue salvage. Yet reperfusion itself triggers a cascade of secondary injurious events (Xiong et al., 2022). These events account for a large share of final neuronal loss. Decades of preclinical work have yielded few approved neuroprotectants, and the approved agents give limited benefit (Tao et al., 2020; Xie et al., 2024). Recent trials of nerinetide, a leading candidate, illustrate this difficulty (Hill et al., 2020, 2025). A persistent gap therefore separates mechanistic understanding from effective therapy. Most research has concentrated on downstream death programs, including ferroptosis, pyroptosis, apoptosis, and necroptosis (Tuo et al., 2022; Zhang et al., 2022). Each is a terminal execution pathway rather than an upstream decision point. How neurons integrate the converging stressors of ischemia and reperfusion remains unclear. How they commit to survival or death before these programs engage is also insufficiently understood.

Recent advances in cell biology have reframed liquid-liquid phase separation (LLPS) and the membraneless organelles it generates. These condensates are not passive bystanders of stress (Banani et al., 2017). They act as regulatory platforms that shape gene expression, protein homeostasis, and signaling (Gerlich, 2017; Shin and Brangwynne, 2017). Among them, stress granules (SGs) are especially relevant to CIRI. Energy deprivation, an oxidative burst, and translational collapse drive rapid SG assembly around scaffold proteins such as G3BP1 (Protter and Parker, 2016). These granules concentrate stalled messenger ribonucleoprotein complexes together with RNA-binding proteins including TDP-43, FUS, and DDX3X. SGs are not inert storage depots. They sequester executioner caspases, support GPX4 expression through an m6A-dependent hub, and modulate inflammatory signaling. Each function carries direct consequences for neuronal fate. Aberrant phase transitions of these same RNA-binding proteins are linked to neuronal disease (Wolozin and Ivanov, 2019). That literature has centered on chronic neurodegenerative disorders, where the relevant time frame spans months to years and the RNA-binding proteins of interest are studied largely apart from the acute metabolic and oxidative perturbations of ischemia. CIRI poses a distinct problem. SG dynamics unfold over hours to days and span two mechanistically different injury phases. They also intersect with death programs, ferroptosis and pyroptosis foremost among them, that have no counterpart in the chronic neurodegeneration literature. A synthesis organized specifically around this acute time frame and this set of downstream pathways has not, to our knowledge, been assembled. The physical state of the condensate, whether reversible droplet or irreversible aggregate, sets its protective or pathological role.

This review examines how LLPS and SG dynamics determine neuronal fate during CIRI. It draws on oxygen-glucose deprivation models, rodent middle cerebral artery occlusion studies, and related neurological conditions where relevant. The account begins with the biophysical principles of LLPS and the molecular architecture of SGs. It gives particular attention to features that make neurons sensitive to phase transition dysregulation. It then examines how ischemia and reperfusion trigger SG assembly, separating the contribution of each phase. The bifurcating fate of SGs under CIRI receives the closest attention. This covers the protective trajectory of reversible liquid condensates and the pathological trajectory of liquid-to-solid transition. The discussion closes with the therapeutic implications of targeting SG dynamics, alongside an honest appraisal of current limitations.

## 2 Biophysical foundations of phase separation and stress granules in neuronal biology

### 2.1 Physical drivers of liquid-liquid phase separation

Liquid-liquid phase separation is a thermodynamic process. A homogeneous solution demixes into two coexisting liquid phases, one dilute and one condensed, once the concentration of participating molecules passes a critical threshold (Spruijt, 2023). In biological systems, proteins with Intrinsically Disordered Regions (IDRs) or Low-Complexity Domains (LCDs) drive this process. These sequences lack stable tertiary structure. They engage in weak, transient, and multivalent interactions with one another and with RNA. The dominant forces include π-π stacking between aromatic residues such as phenylalanine and tyrosine, cation-π attractions between arginine-rich stretches and aromatic rings, and electrostatic contacts between charged patches (Surini and De Curtis, 2026). Because each interaction is weak, the condensate forms and dissolves reversibly as local concentration changes.

One property is especially relevant to CIRI. LLPS is highly sensitive to intracellular physicochemical conditions. Modest drops in pH, shifts in ionic strength, and changes in redox balance each modulate the saturation concentration at which phase separation occurs. ATP exerts a more complex influence. At millimolar levels it acts as a hydrotrope that keeps disordered proteins soluble, so its effect on phase separation is non-monotonic (Patel et al., 2017; Mehringer et al., 2021). This sensitivity has a direct implication. The metabolic and oxidative perturbations of ischemia and reperfusion are not coincidental stressors. They are, in principle, direct modulators of condensate behavior. The LLPS framework therefore explains why neurons respond to CIRI with rapid changes in their membraneless organelle landscape.

### 2.2 Molecular architecture of stress granules

Stress granules are cytoplasmic, membraneless condensates. They assemble when conditions stall translational initiation (Do et al., 2020). They are distinct from processing bodies (P-bodies), which mainly hold transcripts destined for decay. The canonical trigger for SG nucleation is phosphorylation of the alpha subunit of eukaryotic initiation factor 2 (eIF2α). Four stress-responsive kinases can carry this out, namely HRI, PKR, PERK, and GCN2, each responding to a distinct signal (Guo et al., 2020). Phosphorylated eIF2α lowers the availability of the ternary complex needed for translation initiation. Ribosomes then dissociate from messenger RNAs and generate pools of stalled mRNP complexes. These complexes are the raw material from which SGs are built. It is worth emphasizing that eIF2α phosphorylation, translational arrest, and SG formation are closely related but not strictly equivalent processes. Translational stalling supplies the raw material for SGs, but this is not by itself sufficient for their assembly. SGs can also nucleate through eIF2α-independent routes, for example when translation initiation is blocked directly at eIF4A or eIF4F rather than through eIF2α phosphorylation.

**Figure 1:**
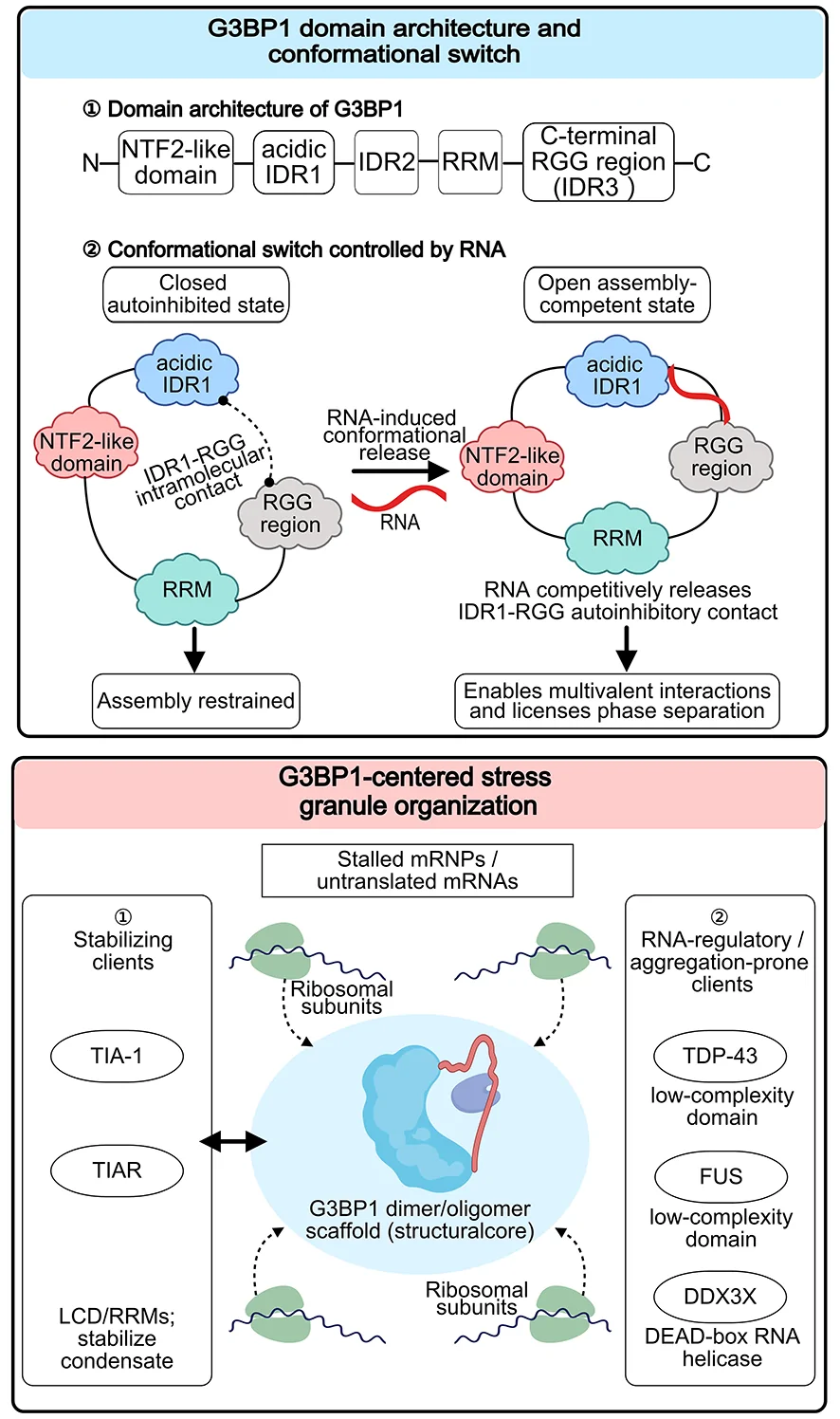
Molecular architecture of stress granules and the G3BP1 conformational switch. G3BP1 contains an NTF2-like domain, acidic IDR1, IDR2, RRM, and C-terminal RGG region. An intramolecular IDR1-RGG contact normally keeps G3BP1 autoinhibited. RNA binding competitively releases this contact, licensing the multivalent interactions that drive assembly. The resulting scaffold organizes stalled mRNPs and ribosomal subunits alongside two client classes. Stabilizing clients such as TIA-1 and TIAR help maintain the condensate. RNA-regulatory, aggregation-prone clients such as TDP-43, FUS, and DDX3X link granule dynamics to neuronal fate in CIRI through their sequestration and eventual mislocalization or aggregation.

Assembly of these stalled complexes into a coherent condensate depends on scaffold proteins. G3BP1 and its paralog G3BP2 are the best characterized scaffolds, and G3BP1 nucleates assembly through multivalent interactions (Yang et al., 2020). It contains several functional elements. These are an NTF2-like domain, an acidic IDR, an RNA recognition motif, and a C-terminal arginine-glycine-rich region that binds RNA. The NTF2-like domain mediates protein-protein contacts, and crystallographic work shows it recognizes specific FxFG repeat motifs. RNA binding to the arginine-glycine-rich region drives a conformational switch that releases autoinhibition and licenses phase separation (Guillén-Boixet et al., 2020). These interactions establish the structural backbone of the nascent SG. Around this scaffold, the granule recruits a diverse set of client proteins. TIA-1 and TIAR contribute LCD-mediated interactions and help stabilize the condensate. TDP-43, FUS, and DDX3X each carry their own IDR and bring specific RNA-regulatory functions into the granule. In neurons these client proteins are abundant and indispensable for normal RNA metabolism (Piol et al., 2023; Pang et al., 2026). Their dysregulation within SGs is therefore especially consequential for cell viability. Table 1 summarizes these proteins together with their normal functions, their roles within the granule, and their consequences in CIRI. Figure 1 shows the domain organization of G3BP1, the RNA-induced conformational switch that licenses assembly, and the arrangement of stabilizing and aggregation-prone clients around the scaffold

**Table 1:** Key proteins of stress granules and their consequences in CIRI.

Protein	Domain or feature	Normal function	Role in stress granule	Consequence in CIRI	References
G3BP1	NTF2-like domain, acidic IDR, RRM, C-terminal RGG region	RNA metabolism and signaling	Scaffold that nucleates assembly through multivalent interactions and an RNA-induced conformational switch	Anchors SG formation, reperfusion modification may shift it toward a less dynamic state	Guillén-Boixet et al., 2020; Yang et al., 2020
TIA-1	Low-complexity domain and RNA recognition motifs	Translational regulation	Client that stabilizes the condensate through its low-complexity domain	Promotes protective SG assembly, molecular target of muscone	Sun et al., 2024
TDP-43	Glycine-rich C-terminal low-complexity domain	Predominantly nuclear, mediates pre-mRNA splicing and cryptic exon suppression	Aggregation-prone client	Cytoplasmic mislocalization after nuclear pore damage, drives solidification	Chou et al., 2018; Bian et al., 2025
FUS	N-terminal low-complexity domain	RNA regulation and local synthesis of synaptic proteins	Highly aggregation-prone client	Progressive cytoplasmic aggregation under oxidative stress, drives solidification and autophagy dysregulation	Monahan et al., 2017; Wu et al., 2022
DDX3X	DEAD-box RNA helicase	RNA metabolism, promotes NLRP3 inflammasome assembly	Client sequestered into liquid-state granules	Sequestration reduces availability for NLRP3, limiting inflammatory signaling	Samir et al., 2019; Guo et al., 2026
IGF2BP1	m6A reader protein	Stabilizes m6A-modified transcripts	Recruited to G3BP1 condensates, forms an epitranscriptomic hub	Upregulates GPX4 protein through the hub, limiting ferroptosis	Li et al., 2026

### 2.3 The liquid-to-solid transition spectrum

The physical state of a stress granule is not fixed. It exists along a spectrum. One end is a fully liquid, dynamically exchanging droplet. The other is an amyloid-like, irreversibly solidified aggregate (López-Erauskin et al., 2018; Ishiguro et al., 2021; Navalkar et al., 2025). Under moderate or transient stress, SGs stay liquid. Their constituent proteins exchange rapidly between the condensed and dilute phases, as shown by fluorescence recovery after photobleaching. This fluidity is functionally meaningful. It allows SGs to disassemble promptly once the initiating stress resolves, releasing sequestered transcripts and restoring normal translation.

These physical states are not simply descriptive labels. They correspond to operational criteria used across the condensate biology literature, although these criteria are not applied uniformly by every study cited in this review. A liquid-state granule is typically defined by rapid and near-complete fluorescence recovery after photobleaching, indicating free exchange of its components with the surrounding cytoplasm (Patel et al., 2015). It also shows the capacity of separate droplets to fuse on contact and to disassemble fully once the inciting stress is removed. A gel-like granule shows partial and slowed internal mobility, incomplete or delayed FRAP recovery, and reduced fusion capacity, marking an intermediate state along the spectrum. A solid-like or irreversible aggregate shows minimal or absent FRAP recovery, loss of fusion capacity, and resistance to disassembly after stress removal. It is often further characterized biochemically as detergent-insoluble material recovered in a high-speed pellet fraction or as amyloid-like structure detected by thioflavin-T or similar dyes. Few of the primary studies discussed below report the full panel of these measurements for the same condensate. This review therefore uses “liquid,” “gel-like,” and “solid-like” as descriptive terms following the usage of the cited primary studies, not as claims that each instance meets every criterion above.

A related distinction concerns the level at which a phase transition is assessed. The detection or colocalization of an aggregation-prone client protein such as TDP-43 or FUS within a granule does not, by itself, establish that the granule as a whole has solidified. A client protein can shift toward a less dynamic state while the bulk condensate, including the G3BP1 scaffold, remains comparatively fluid. The converse is also possible. TDP-43 and FUS are, moreover, each capable of forming condensates or aggregates independently of canonical SGs altogether, through direct nuclear-to-cytoplasmic mislocalization or self-assembly outside the SG pathway (Gasset-Rosa et al., 2019). Throughout this review, granule-level solidification and client-protein aggregation are therefore treated as related but distinguishable phenomena. Where the underlying study allows it, the text specifies which of the two was actually measured.

Sustained or severe stress drives a different outcome. SGs then undergo a process called aging. Internal mobility progressively falls, protein interactions become less reversible, and the condensate shifts toward a gel-like or solid state. Post-translational modifications accelerate this aging. These include oxidation, aberrant phosphorylation, and the buildup of misfolded protein species within the granule (Gui et al., 2019; Li et al., 2022). The distinction between liquid and solid states is not merely biophysical. It carries direct biological consequences. Reversible liquid-state SGs are broadly cytoprotective. Solid-state aggregates are associated with proteostasis failure and cell death. This spectrum is the conceptual foundation for understanding one duality of CIRI. The same machinery that initially protects neurons can, under prolonged injury, drive irreversible damage. Later discussion examines this duality in detail.

A caveat applies to this framework as a whole. Much of the biophysical and structural evidence summarized above was generated in cell-free reconstitution systems, immortalized cell lines, or non-neuronal contexts rather than in primary neurons or intact brain tissue. This includes the thermodynamic principles of LLPS, the domain-level dissection of G3BP1, and the FRAP-based criteria proposed for classifying condensate states. Neurons carry structural features such as extended processes, polarized transport, and a prolonged post-mitotic lifespan. These features could plausibly shift the thermodynamics or kinetics of phase separation relative to the systems in which these principles were established. This section is therefore best read as the mechanistic vocabulary available for describing SG behavior in CIRI, not as a body of evidence already validated in that setting. The sections that follow return repeatedly to how much of it has, and has not, been confirmed directly in ischemic neurons.

## 3 Molecular and temporal control of stress granule assembly in CIRI

### 3.1 eIF2α-dependent nucleation during the ischemic phase

**Figure 2:**
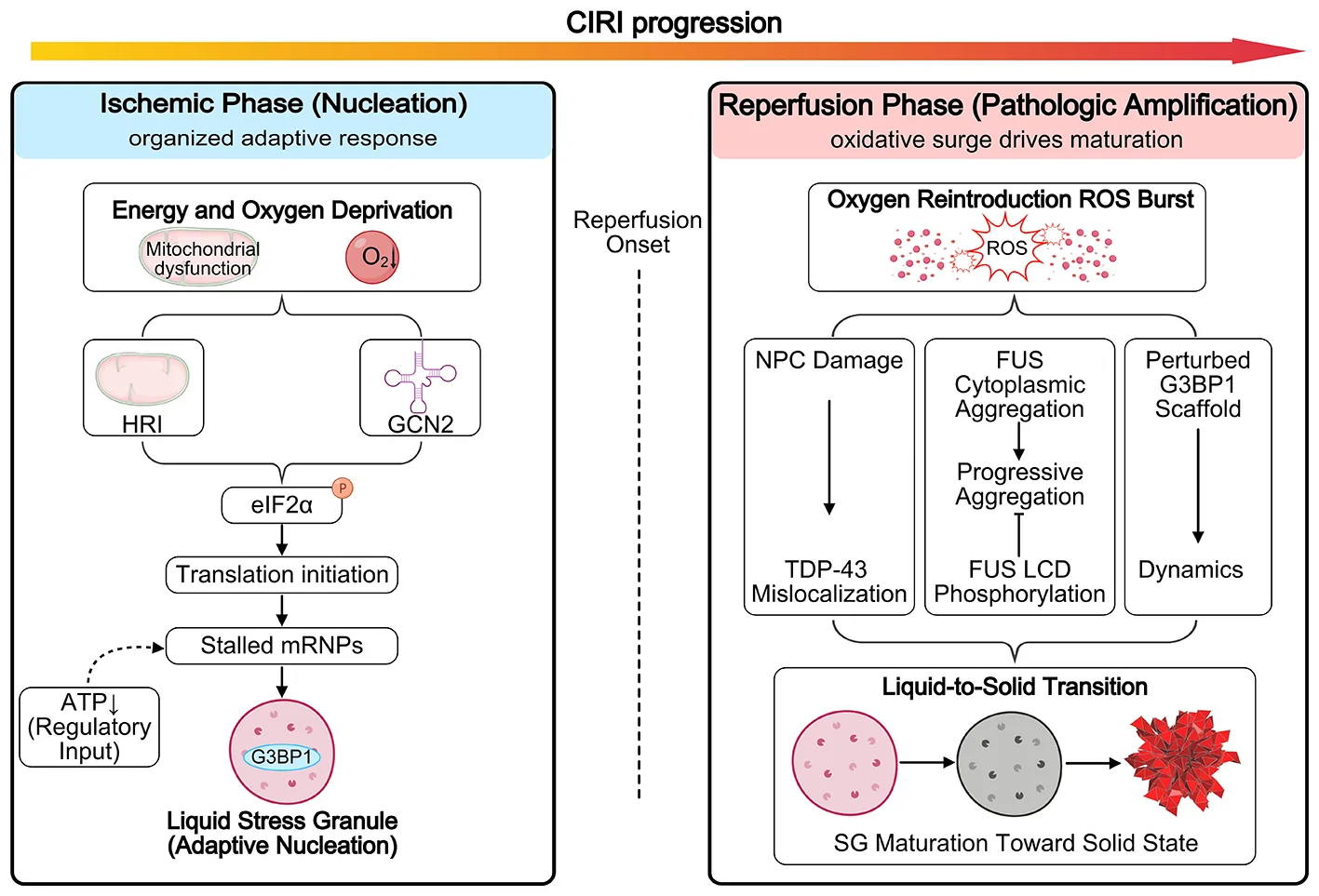
Two-phase assembly of stress granules during cerebral ischemia-reperfusion injury. During ischemia **(left)**, energy and oxygen deprivation activate the stress kinases HRI and GCN2, driving eIF2α phosphorylation, translational arrest, and adaptive nucleation of a liquid stress granule around G3BP1. Falling ATP acts as an additional, non-monotonic modulator of this assembly. During reperfusion **(right)**, an oxidative burst acts on three fronts. These are nuclear pore damage promoting TDP-43 mislocalization, sustained oxidative stress driving progressive FUS aggregation, and perturbation of G3BP1 scaffold dynamics. Together, these changes mature the granule from a liquid toward a gel-like and then solid state.

Stress granule assembly in CIRI is not a random response to damage. It reflects a conserved and molecularly precise stress-sensing machinery. Figure 2 outlines this two-phase progression, contrasting adaptive nucleation in ischemia with pathological amplification in reperfusion. Under oxygen-glucose deprivation (OGD), the integrated stress response (ISR) engages eIF2α through a set of four kinases, namely HRI, PKR, PERK, and GCN2 (Costa-Mattioli and Walter, 2020; English et al., 2022). Two are especially relevant to ischemia. HRI, the heme-regulated inhibitor kinase, responds to mitochondrial dysfunction and the reactive oxygen species of early ischemia. GCN2 responds to the uncharged transfer RNAs that accumulate under amino acid shortage. Both converge on phosphorylation of eIF2α at serine 51. This reduces the eIF2-GTP-Met-tRNA ternary complex and stalls translational initiation across the transcriptome.

Translational arrest produces stalled mRNP complexes in the cytoplasm. These complexes condense around G3BP1 nucleation sites in a concentration-dependent manner. The biophysical shifts of ischemia facilitate this process. ATP is one relevant variable. Beyond its metabolic role, ATP at millimolar levels acts as a hydrotrope, so its influence on phase separation is non-monotonic. Ren and colleagues showed that ATP produces reentrant phase behavior in FUS solutions (Ren et al., 2022). In their analysis, condensates dissolved only as ATP approached roughly 10 mM. ATP depletion during ischemia therefore reshapes condensate behavior in a manner that depends on the concentration regime. The rapid assembly seen within minutes of ischemic onset is driven mainly by kinase-mediated arrest and the resulting pool of stalled mRNPs. Shifting ATP acts as one modulating influence among several. This initial response is an active and organized adaptation, not a marker of impending failure. By concentrating stalled transcripts and sequestering specific RNA-binding proteins, neurons pause non-essential translation. They retain the capacity to synthesize stress-response proteins whose mRNAs carry upstream open reading frames that resist eIF2α-mediated inhibition.

### 3.2 Oxidative modification of stress granule components during the reperfusion phase

Ischemia establishes the initial conditions for SG formation. Reperfusion introduces a different set of insults that act directly on SG components. These insults drive the shift from a reversible liquid condensate toward a pathological solid aggregate. The burst of reactive oxygen species on oxygen reintroduction far exceeds that of the ischemic phase. This surge reshapes SG components in two ways. It relocalizes aggregation-prone RNA-binding proteins into the cytoplasm, and it activates kinases that modify phase behavior within the granule.

The scaffold protein G3BP1 remains relevant to this process. Its contribution is governed less by direct oxidation than by changes in its non-covalent assembly properties. G3BP1 dimerizes through its NTF2-like domain, which also recruits partners such as Caprin1 and USP10 (Kedersha et al., 2016). The RNA-gated conformational switch described earlier also organizes competing protein-RNA interaction networks within the granule (Sanders et al., 2020). A separate and older model proposed that phosphorylation of G3BP1 at Ser149 regulates SG assembly. Tourrière and colleagues advanced this view, and Reineke and colleagues later linked casein kinase 2 activity at this residue to granule dynamics (Reineke et al., 2017). This phosphosite model remains contested. Panas and colleagues re-examined the question and found that this phosphorylation does not control SG assembly (Panas et al., 2019). Under the oxidative and metabolic conditions of reperfusion, perturbation of these inputs may shift the scaffold toward a less dynamic state. The precise determinants in CIRI remain to be defined.

One principal consequence of the oxidative surge is disruption of nucleocytoplasmic transport. Oxidative injury to nuclear pore complexes compromises the barrier that normally retains TDP-43 in the nucleus. TDP-43 then accumulates in the cytoplasm, where its glycine-rich C-terminal low-complexity domain enters the SG environment. There it engages in aggregation-prone interactions that accelerate the liquid-to-solid transition. Bian and colleagues documented the timing in a transient MCAO model (Bian et al., 2025). Cytoplasmic TDP-43-positive cells and the G3BP1 signal both peaked at 6 h after reperfusion. The G3BP1 signal declined by 24 h. The proteasomal shuttling factor RAD23B co-localized with TDP-43 later, at 24 h, alongside upregulated HDAC6. This offset places engagement of the proteostasis machinery after the early peak of SG assembly rather than concurrent with it. Reperfusion therefore actively shapes the cytoplasmic handling of an aggregation-prone protein.

FUS follows a related route. It joins SGs through its N-terminal low-complexity domain and is among the most aggregation-prone proteins in the granule. As reperfusion sustains oxidative stress, FUS partitions increasingly into cytoplasmic SGs, and the conditions for its transition accumulate. A phosphorylation-based safeguard exists. Phosphorylation of the FUS low-complexity domain adds negative charge that increases electrostatic repulsion and opposes aggregation. Under sustained CIRI, this safeguard is outpaced by the rising aggregation pressure.

### 3.3 Neuronal vulnerability to stress granule solidification

The molecular events above unfold across multiple cell types within the ischemic territory. Yet neurons show a disproportionate vulnerability to the consequences of SG solidification. Several converging factors account for this disparity. TDP-43, FUS, and DDX3X are abundant in neurons, but they are broadly expressed across the central nervous system and are not confined to neurons (Nowakowski et al., 2017). The vulnerability of neurons reflects their reliance on these factors rather than a higher absolute concentration. Because these proteins are essential for neuronal RNA splicing, transport, and local translation, their sequestration deprives neurons of factors that cannot be readily replaced. Non-neuronal cells carry greater transcriptional plasticity and higher baseline chaperones such as HSP70 and DNAJB1 (Coleman et al., 2026). This provides a buffer against condensate aging that neurons lack.

Post-mitotic neurons also operate with constrained proteasomal and autophagic throughput compared with proliferating glia (Pytel and Fromm Longo, 2025). Under normal conditions, efficient quality control manages this limit. When solid-phase SG aggregates present as competing substrates, the available degradative capacity is rapidly saturated. Failed clearance then drives a feed-forward cycle. Aggregated proteins recruit additional soluble clients into the insoluble fraction, depleting the functional pool of essential RNA-binding proteins.

A further factor is the compartmentalized nature of neuronal translation. Local synthesis in axons and dendrites depends on the transport and on-site assembly of mRNP complexes (Naskar et al., 2023). Many of these share compositional features with SG material. When reperfusion-driven modifications render distal SG-like assemblies resistant to disassembly, the clearance machinery must act across geometrically unfavorable distances. Peripheral neuronal compartments are therefore particularly exposed. Observations by Xu and colleagues in brain vascular endothelial cells show that TDP-43 fragments alter endothelial signaling (Xu et al., 2022). This is not an isolated finding. The same endothelial compartment develops refractory, solid-phase SGs after prolonged ischemia and delayed recanalization, and astrocytes accumulate solidified FUS with a dysregulated autophagic response after MCAO (Wu et al., 2022; Lu et al., 2023). SG pathology across the neurovascular unit therefore extends well beyond neurons. Because endothelial integrity and astrocytic support functions are themselves prerequisites for neuronal survival, solidification in these compartments may compound neuronal injury rather than proceed independently of it.

The comparative claim that neurons are disproportionately vulnerable is nonetheless built on indirect evidence. No study identified here has measured SG solidification side by side in neurons, endothelial cells, and astrocytes within the same CIRI model. The neuronal data, the endothelial data, and the astrocytic data come from separate experiments with different injury durations and readouts (Wu et al., 2022; Xu et al., 2022; Lu et al., 2023; Bian et al., 2025). The argument above for neuronal vulnerability rests on reliance on irreplaceable RNA-binding proteins, constrained degradative throughput, and compartmentalized translation. It is therefore a plausibility argument grounded in known neuronal cell biology rather than a demonstrated comparison. It should be read as identifying where solidification is likely to have the most severe consequences once it occurs, rather than as evidence that neurons solidify first, fastest, or exclusively.

### 3.4 The dual fate of stress granules in CIRI

**Figure 3:**
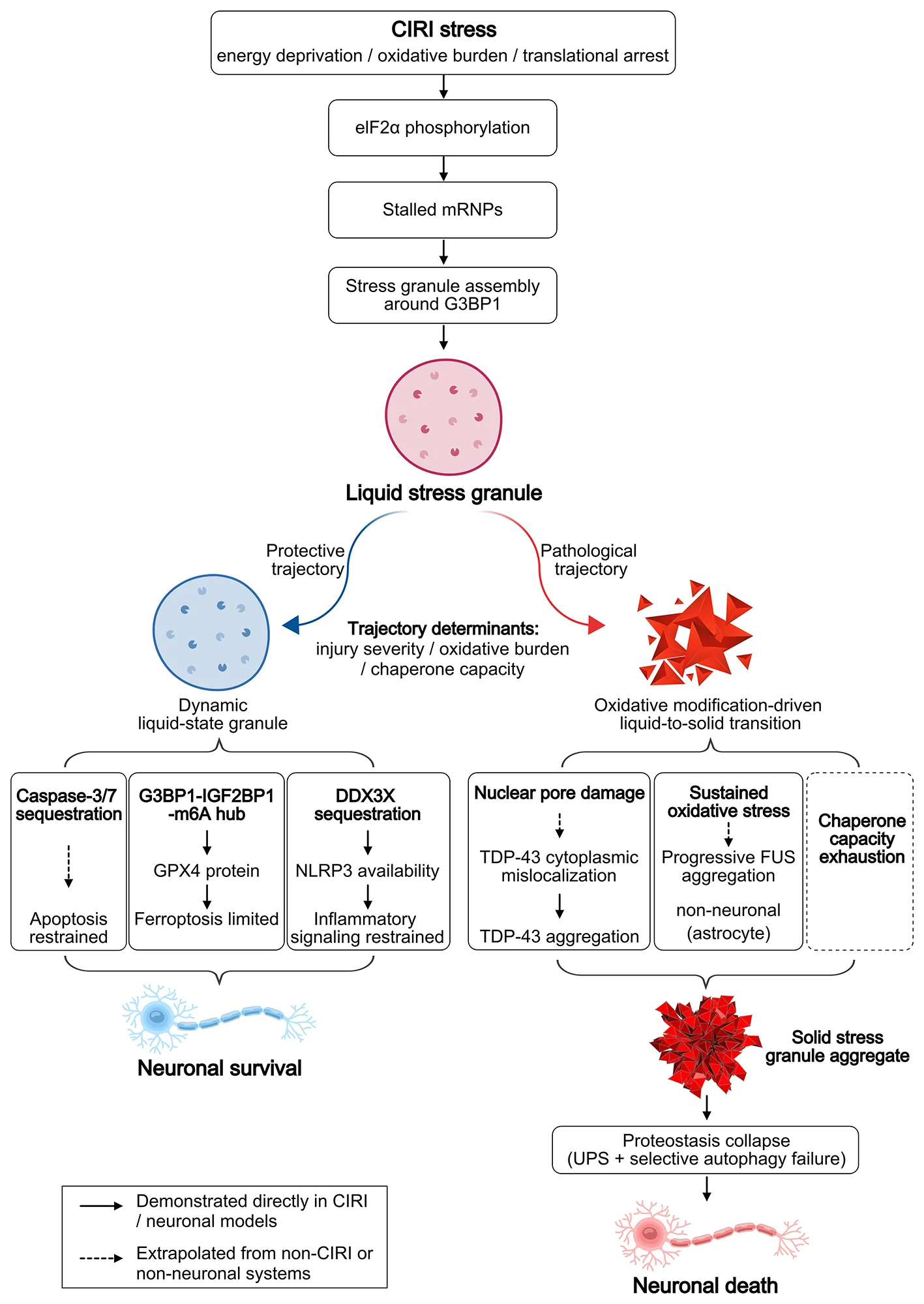
Stress granule physical state as a determinant of neuronal fate in cerebral ischemia-reperfusion injury. CIRI stress triggers eIF2α phosphorylation and stalled mRNP accumulation, nucleating a liquid-state stress granule around G3BP1. Injury severity, oxidative burden, and chaperone capacity determine whether the granule follows a protective or pathological trajectory. Solid arrows mark mechanisms demonstrated directly in CIRI and/or neuronal models. Dashed arrows mark mechanisms extrapolated from non-CIRI or non-neuronal systems. Liquid-state granules restrain apoptosis, ferroptosis, and inflammatory signaling. Oxidative modification instead drives liquid-to-solid transition, TDP-43 and FUS aggregation, proteostasis collapse, and neuronal death.

The events described above establish that SG assembly is an early and consistent neuronal response to CIRI. What happens to these condensates after they form has received far less attention. Yet that outcome determines whether the neuron survives or dies. Evidence from recent years points to a bifurcating trajectory. Figure 3 presents this bifurcation as a whole, from SG assembly through the protective and pathological trajectories to neuronal survival or death. Under moderate or timely-resolved injury, SGs stay liquid and dynamic and confer measurable cytoprotection through at least three distinct mechanisms. Under sustained or severe injury, the same condensates solidify irreversibly, disrupt proteostasis, and commit the neuron to death. These trajectories are not quantitative variants of one process. They are qualitatively different outcomes driven by distinguishable molecular events. The discussion addresses the protective trajectory first, then the pathological one, before examining the factors that determine which path a neuron follows. Table 2 summarizes the primary studies that inform both trajectories, together with the model used and the type of evidence each provides.

**Table 2:** Primary studies linking stress granule dynamics to neuronal fate in ischemic models.

Model	Key findings	Stress granule mechanism	Evidence type	References
Mice, transient MCAO of 30 or 60 min, examined at 1, 6, and 24 h after reperfusion	TDP-43 and G3BP1 peaked at 6 h. RAD23B colocalized with TDP-43 at 24 h.	SG assembly and TDP-43 mislocalization precede proteostasis engagement	Direct, CIRI, neuronal (*in vivo*)	Bian et al., 2025
Brain endothelial cells and MCAO	TDP-43 C-terminal fragment rose 24 to 72 h after MCAO and altered tight junction and MST1/2-YAP signaling.	Cytoplasmic TDP-43 fragments alter endothelial signaling	Direct, CIRI, non-neuronal (endothelial)	Xu et al., 2022
Bone marrow-derived macrophages, arsenite and LPS plus nigericin	DDX3X drives NLRP3 activation. SG assembly sequesters DDX3X and inhibits pyroptosis.	SG sequestration of DDX3X restrains NLRP3, setting a rheostat between survival and inflammatory death	Conceptual source, non-CIRI (macrophages)	Samir et al., 2019
Sprague-Dawley rats, remote ischemic conditioning initiated 6 h after reperfusion, with sequential exercise	Conditioning increased SGs, reduced DDX3X, attenuated NLRP3, and lowered neuronal death.	Preserved SG integrity reduces DDX3X availability and restrains NLRP3	Indirect, associative (causal chain in neurons not directly shown)	Wang et al., 2024
OGD/R cell models and MCAO rats	Icariin bound G3BP1 and promoted SG assembly. GPX4 protein rose and infarct volume fell, both G3BP1 and IGF2BP1 dependent.	G3BP1-IGF2BP1-m6A hub upregulates GPX4 protein and limits ferroptosis	Direct, CIRI (*in vitro* and *in vivo*)	Li et al., 2026
PC12 cells under OGD/R and MCAO/R rats	Muscone bound TIA1 and promoted SGs, reducing apoptosis and infarct volume.	TIA1-dependent SG assembly is cytoprotective	Direct, CIRI (*in vitro* and *in vivo*)	Sun et al., 2024
Rats, 60-min MCAO and reperfusion	Rapamycin reduced insoluble TDP-43 and lowered apoptosis and infarct volume.	Autophagic flux opposes TDP-43 solidification	Direct, CIRI (*in vivo*)	Tsou et al., 2022
Mice, MCAO	FUS aggregation in astrocytes drove excessive autophagy and aggravated damage.	Solidified FUS perturbs autophagy regulation in astrocytes	Direct, CIRI, non-neuronal (astrocyte)	Wu et al., 2022
Brain vessel endothelial cells, prolonged ischemia and delayed recanalization	Melatonin resolved refractory solid SGs and ameliorated ER stress, giving dual-phase protection.	Resolution of refractory solid SGs restores endothelial function	Direct, CIRI, non-neuronal (endothelial)	Lu et al., 2023

### 3.5 The protective trajectory of liquid-state stress granules

#### 3.5.1 Sequestration of executioner caspases restrains apoptosis initiation

One of the most direct protective mechanisms is the physical sequestration of executioner caspases into the condensate. Fujikawa and colleagues mapped the SG proteome using proximity-dependent biotin labeling anchored on G3BP1 and TIA1 (Fujikawa et al., 2023). They identified caspase-3 and caspase-7 as resident SG components. Recruitment depends on conserved residues within their large catalytic domains. Accumulation inside the granule lowers caspase activity and suppresses apoptosis induced by diverse stresses. An SG-localization-deficient caspase-3 mutant abolished much of this effect. Forced relocalization of that mutant back to SGs restored protection. This establishes the sequestration itself as the causal event rather than a correlate. Caspase-3 is the principal executioner protease in neuronal apoptosis after ischemia-reperfusion. Partitioning active caspase pools into liquid-state granules therefore raises the threshold for apoptotic commitment during the acute phase. This mechanism was established in cancer cell lines and a xenograft model rather than in ischemic neurons. Its operation in CIRI is a reasonable extension that awaits direct confirmation. In parallel, the eIF2α phosphorylation that nucleates SGs reprograms translation. This permits synthesis of ATF4 through its upstream open reading frames, supporting the adaptive redox and amino acid programs active while granules remain liquid (Bohlen et al., 2020; Adjibade et al., 2024; Huang et al., 2026).

#### 3.5.2 Recruitment of the m6A reader IGF2BP1 upregulates GPX4 and suppresses ferroptosis

A mechanistically distinct function involves upregulation of glutathione peroxidase 4 (GPX4) through an epitranscriptomic route. IGF2BP1 is a recognized reader of N6-methyladenosine (m6A) modifications. During CIRI it is recruited to G3BP1 condensates, where it forms a hub that engages m6A-modified survival transcripts and drives downstream m6A-dependent signaling. Li and colleagues demonstrated this mechanism using icariin in OGD/R and MCAO models (Li et al., 2026). Icariin raised GPX4 protein after OGD/R, and this increase was abolished by G3BP1 knockdown and by IGF2BP1 knockdown. The authors framed the rise as an output of an m6A-dependent AMPK-MAPK-GPX4 axis. GPX4 is the principal enzymatic suppressor of lipid peroxidation and the node at which ferroptotic signaling is restrained (Stockwell et al., 2017). Its upregulation during the acute phase therefore forms a meaningful barrier against iron-dependent death. The finding bridges condensate biology with one of the most actively studied death programs in the ischemic brain.

#### 3.5.3 DDX3X sequestration limits NLRP3-dependent inflammatory signaling

A third function is mediated by the RNA helicase DDX3X, recruited to SGs as a client during CIRI. The mechanistic basis was established by Samir and colleagues in macrophages rather than neurons (Samir et al., 2019). They showed that DDX3X binds the NACHT domain of NLRP3 and drives inflammasome activation. SG assembly then sequesters DDX3X and reduces NLRP3 activation, ASC speck formation, and pyroptosis. The two compartments compete for a shared pool of DDX3X, which sets a rheostat between survival and inflammatory death. This concept was developed in myeloid cells, so its initial application in the ischemic brain was an extrapolation rather than a demonstrated fact. The extrapolation is biologically motivated, since NLRP3 activation in the ischemic brain drives caspase-1-dependent pyroptosis and the release of interleukin-1β. Wang and colleagues provided indirect support in a CIRI setting (Wang et al., 2024). Early remote ischemic conditioning, applied during reperfusion, preserved SG integrity in neurons and was associated with attenuated NLRP3 activation. This association is consistent with SGs acting upstream of inflammasome restraint, and more direct biochemical evidence has since followed. Guo and colleagues used co-immunoprecipitation in whole brain tissue from a rat MCAO model to show that reperfusion strengthens the DDX3X-NLRP3 interaction. Normobaric oxygen therapy reverses this by strengthening the competing DDX3X-G3BP1 interaction, which lowers NLRP3 activation and pyroptotic markers (Guo et al., 2026). This places the DDX3X-SG-NLRP3 rheostat on direct biochemical footing in the ischemic brain for the first time, rather than as a pure extrapolation from myeloid cells. The evidence is still tissue-level rather than specificity-isolated. It comes from whole-brain lysate rather than a neuron-specific readout. No study has yet used a SG-assembly-deficient control of the kind that isolates the GPX4 effect of icariin, described above.

### 3.6 The pathological trajectory of liquid-to-solid transition

#### 3.6.1 TDP-43 mislocalization drives irreversible cytoplasmic aggregation

When CIRI extends beyond the threshold that liquid SGs can accommodate, aggregation-prone species progressively reshape the condensate. TDP-43 is the most consequential of these in neurons. Under normal conditions, TDP-43 is predominantly nuclear, where it mediates pre-mRNA splicing, transcript stability, and cryptic exon suppression (Rummens and Da Cruz, 2025). Its nuclear retention depends on intact nuclear pore complexes. Reperfusion-associated oxidative damage impairs this barrier, and TDP-43 redistributes to the cytoplasm, where it meets the SG environment established during ischemia. The link between nuclear pore damage and TDP-43 mislocalization is established in ALS and frontotemporal dementia (Chou et al., 2018). Its operation in CIRI is therefore a well-motivated extrapolation rather than a directly demonstrated finding. Notably, cytoplasmic TDP-43 can also de-mix independently of stress granules and inhibit nuclear import on its own, which indicates that SG entry is one route to toxicity rather than the only one (Gasset-Rosa et al., 2019).

Once cytoplasmic, TDP-43 engages through its glycine-rich C-terminal low-complexity domain. This region is essential for RNA regulation and prone to amyloid-like contacts when molecular mobility falls. The declining fluidity of an aging SG provides exactly such conditions, and TDP-43 shifts from a dynamic client into a committed aggregate nucleus. Bian and colleagues documented features of this process in a transient MCAO model (Bian et al., 2025). Cytoplasmic TDP-43 co-localized with the proteasomal shuttling factor RAD23B at 24 h, alongside upregulated HDAC6, after the 6 h peak of the G3BP1 signal. HDAC6 recruits ubiquitinated misfolded proteins to the aggresome and supports their autophagic clearance (Jo et al., 2022). This co-localization is therefore best read as recruitment of the proteostasis machinery to mislocalized TDP-43 rather than evidence that HDAC6 stabilizes the aggregates. Separately, Tsou and colleagues showed that rapamycin reduced insoluble TDP-43 in MCAO rats (Tsou et al., 2022). This implicates autophagic flux as a counterforce to solidification that is nonetheless insufficient under severe injury. The functional consequence of irreversible aggregation is twofold. Neurons lose the splicing and stability functions TDP-43 normally provides, and the aggregated protein acquires toxic properties, likely including sequestration of further RNA-binding proteins into the insoluble fraction.

#### 3.6.2 FUS aggregation disrupts local translation and autophagy

FUS follows a parallel but distinct path toward solidification. Its N-terminal low-complexity domain, rich in glutamine, glycine, serine, and tyrosine, is among the most aggregation-prone sequences in neurodegenerative disease research. Incorporation into SGs during CIRI places it where the conditions for irreversible transition are progressively met. These conditions arise mainly from sustained partitioning of FUS into cytoplasmic condensates and the persistence of oxidative stress, which together mature FUS granules toward a gel-like and then solid state. A phosphorylation-based safeguard opposes this shift. Phosphorylation of the FUS low-complexity domain increases negative charge and electrostatic repulsion, which disrupts aggregation-prone interactions and helps preserve fluidity (Monahan et al., 2017). FUS solidification under severe CIRI therefore reflects a setting in which aggregation pressure exceeds the capacity of this and other safeguards.

Wu and colleagues observed that FUS aggregation in astrocytes after MCAO was associated with a dysregulated autophagic response (Wu et al., 2022). Autophagy was activated beyond the level that promotes selective clearance and instead contributed to dysfunction. This observation was made in astrocytes rather than neurons. Even so, the underlying mechanism, in which solidified FUS disrupts the coupling between autophagy induction and cargo selectivity, is plausible in neurons given the shared machinery. The translational consequences are particularly damaging in neuronal processes, where FUS normally supports the local synthesis of synaptic proteins. When FUS solidifies in axons or dendrites, local translational programs fail in compartments remote from the somatic quality-control machinery, leaving these regions unable to restore protein homeostasis.

#### 3.6.3 Proteostasis collapses through joint proteasome and autophagy failure

The solidification of TDP-43 and FUS does not occur in isolation. It initiates a broader failure of the proteostasis network that amplifies injury. Solid-phase aggregates present as atypical substrates for the ubiquitin-proteasome system, engaging the machinery without being efficiently processed. Under the metabolic constraints of CIRI, proteasomal capacity is already reduced. The added burden of aggregated material leaves canonical substrates, including damaged signaling proteins and misfolded translation factors, to accumulate without clearance.

Selective autophagy is similarly impaired. It would normally resolve SG material through the disaggregase activity of VCP and recognition by receptors such as p62 and NBR1 that bridge ubiquitinated cargo to LC3-decorated autophagosomes (Tamura and Waguri, 2026). This clearance route depends on HDAC6, which controls the autophagosome maturation required for ubiquitin-selective quality control and provides an essential link between autophagy and the ubiquitin-proteasome system (Chang et al., 2021). TDP-43 aggregates are thought to reduce the accessibility of ubiquitin tags and to interfere with efficient p62 oligomerization and LC3 engagement, although the precise basis in CIRI remains to be defined. The result is a feed-forward cycle in which failed clearance drives further accumulation, which deepens proteasomal and autophagic saturation. As this cycle progresses, neurons lose the minimal proteostasis required for survival. Death through apoptosis, ferroptosis, or pyroptosis then follows not from any single trigger but as the downstream outcome of a generalized collapse in protein quality control. That collapse was initiated, and could in principle have been interrupted, at the level of SG solidification.

### 3.7 The time window of trajectory bifurcation

**Figure 4:**
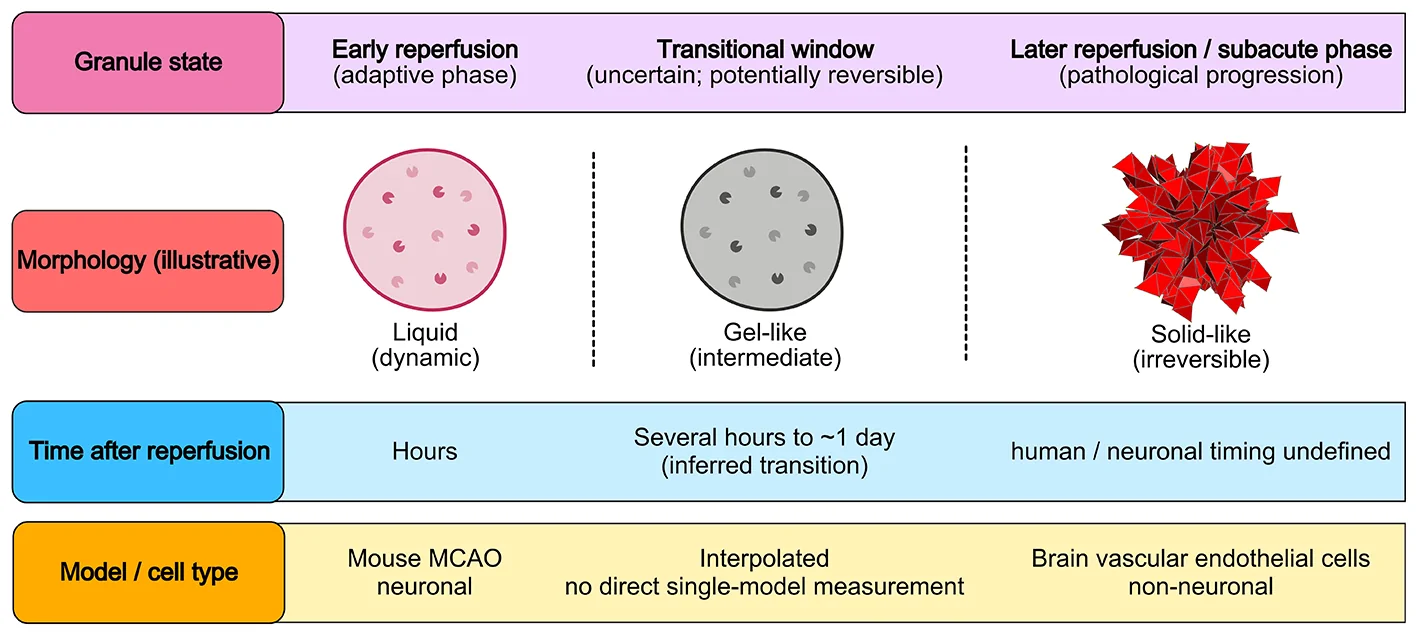
Approximate temporal progression of stress granule solidification during reperfusion. This is a schematic, hypothetical timeline rather than a continuous measurement in a single model. The liquid and solid-like endpoints are anchored to separate studies using different cell types. Liquid-state granules were near their peak approximately 6 hours after reperfusion in neurons from a mouse MCAO model (Bian et al., 2025). Solid-phase granules were observed in brain vascular endothelial cells after prolonged ischemia with delayed recanalization (Lu et al., 2023). The intermediate gel-like state and its ~1-day boundary are interpolated, not directly measured, and the human and neuronal time window remains undefined.

The distinction between the two trajectories is temporal as well as mechanistic. Understanding the window within which the bifurcation occurs is essential for any therapy aimed at shifting neuronal fate toward survival. Evidence from rodent CIRI models suggests that the transition from reversible to irreversible SGs is not instantaneous but develops over the subacute reperfusion phase. Any estimate of its timing is an inference. The available studies differ in cell type, injury duration, and the readout used to define solidification, and they do not converge on a single interval. Lu and colleagues studied brain vessel endothelial cells under prolonged ischemia and delayed recanalization (Lu et al., 2023). They found that the granules had become refractory and solid by the time of recanalization, requiring melatonin to dissolve them. The study did not assign a specific hour to the onset of solidification. Its design indicates only that the transition was already established after prolonged ischemia in that endothelial setting. In neurons, the data of Bian and colleagues show cytoplasmic TDP-43 and the G3BP1 signal peaking near 6 hours after reperfusion, before the peak of neuronal death (Bian et al., 2025). This indicates that SG assembly and the mislocalization of aggregation-prone protein precede terminal death rather than coinciding with it. Read together, these observations point to a transition that begins within the first hours of reperfusion and may take a further day or more to become fully established. The precise timing varies with injury severity and cell type rather than sitting at a fixed hour. Figure 4 places these observations on an approximate timeline and marks the reversible window as an interval rather than a fixed point.

Three categories of biological variable appear to govern where the bifurcation falls for a given neuron. The first is injury severity, reflected in the duration of ischemia and the magnitude of the reperfusion ROS burst. Both determine how rapidly the oxidative modifications that accelerate aging accumulate. The second is molecular chaperone capacity, particularly the levels of HSP70, HSP90, and their co-chaperones. These oppose the formation of irreversible contacts within aging condensates, and their expression declines with age and with metabolic comorbidities such as diabetes. The third is the efficiency of ISR resolution, including the phosphatase complexes that dephosphorylate eIF2α and the disaggregase activity of VCP and related AAA-ATPases that facilitate SG disassembly once stress passes.

Read together, the evidence for this bifurcating model varies considerably in how directly it was obtained in CIRI. Of the three protective mechanisms described above, only the G3BP1-IGF2BP1-m6A-GPX4 axis has been demonstrated directly in ischemic neurons with a causal manipulation. G3BP1 and IGF2BP1 knockdown abolished the protective effect (Li et al., 2026). Caspase sequestration rests on a xenograft and cancer-cell-line model. The DDX3X-NLRP3 rheostat sits in between these two extremes. It originated in macrophage biology. Co-immunoprecipitation in whole brain tissue from a rat MCAO model now shows the same interaction shifting in the predicted direction after reperfusion and under a candidate therapy (Guo et al., 2026). This adds to the earlier associative support from remote ischemic conditioning (Wang et al., 2024). That is tissue-level biochemical evidence in the ischemic brain, not the specificity-isolated demonstration available for the GPX4 axis. Of the two pathological mechanisms described above, the occurrence of TDP-43 mislocalization and FUS aggregation after CIRI is each supported by direct *in vivo* observation (Wu et al., 2022; Bian et al., 2025). The causal mechanisms proposed to drive them are carried over from ALS/FTD research and biophysical work performed outside CIRI. These are nuclear pore damage for TDP-43 and the oxidative override of a phosphorylation-based safeguard for FUS. The claim that solidification precipitates a joint proteasome-autophagy collapse is the most inferential link in the model. It is compatible with the finding that rapamycin reduces insoluble TDP-43 alongside apoptosis and infarct volume (Tsou et al., 2022). That result demonstrates that autophagy modulation affects outcome, but it does not show that solidified SGs are the specific substrate driving proteostasis failure. The bifurcating model is therefore best understood as an integration of several tiers of evidence rather than as a uniformly established pathway. It combines one well-supported CIRI-specific mechanism, one mechanism with emerging tissue-level CIRI support that still lacks specificity isolation, several biologically motivated extrapolations, and one largely inferential downstream link.

### 3.8 Therapeutic strategies targeting stress granule dynamics in CIRI

The framework developed above carries a direct implication for therapy. If SG dynamics occupy an upstream position in the CIRI injury cascade, then interventions that preserve the protective liquid state or prevent pathological solidification should improve neuronal survival. This benefit should hold independently of which downstream death program predominates. The strategies below are organized by the temporal stage at which they act, reflecting the bifurcating trajectory of SG fate. Table 3 summarizes these agents by stage, with their molecular targets, supporting evidence, and limitations.

**Table 3:** Pharmacological agents targeting stress granule dynamics by temporal stage.

Agent	Stage	Target or mechanism	Model evidence	Limitations	References
Muscone	Acute, SG stabilization	Binds TIA1 and promotes SG assembly	PC12 OGD/R and MCAO/R rats	Rodent and cell only, TIA1 has roles beyond SGs	Sun et al., 2024
Icariin	Acute, SG stabilization	Binds G3BP1, drives condensate topology shift, upregulates GPX4 protein via IGF2BP1-m6A hub	OGD/R cell models and MCAO rats	Rodent and cell only, very recent finding awaiting replication	Li et al., 2026
Remote ischemic conditioning	Acute, SG stabilization	Preserves SG integrity, reduces DDX3X availability, restrains NLRP3	Sprague-Dawley rats, conditioning 6 h after reperfusion	Non-pharmacological, neuronal causal chain not directly shown	Wang et al., 2024
Melatonin	Subacute, aggregate resolution	Resolves refractory solid SGs and ameliorates ER stress through enhanced autophagy	Brain vessel endothelial cells, prolonged ischemia and recanalization	Endothelial not neuronal, direct or chaperone-mediated route undefined	Lu et al., 2023
HSP70 induction	Subacute, aggregate resolution	Maintains solubility of TDP-43 and FUS low-complexity domains	ALS and frontotemporal dementia models	No direct *in vivo* evidence in ischemic brain	Gu et al., 2021; Li et al., 2022
HDAC6 inhibition (tubastatin A)	Subacute, aggregate resolution	Restores α-tubulin acetylation and microtubule transport, activates Nrf2 and HO-1 signaling	Rodent MCAO models	HDAC6 also required for aggresome formation and aggregate clearance	Wang et al., 2016; Li et al., 2019; Chang et al., 2021

### 3.9 Pharmacological stabilization of stress granule assembly in the acute phase

The rationale for promoting SG assembly in the acute phase rests on the earlier evidence that the initial condensate response is cytoprotective. Enhancing or sustaining this response while SGs remain liquid can amplify its benefits. Three interventions have been reported to operate through this mechanism, with varying mechanistic specificity.

Muscone is a macrocyclic ketone derived from musk. Sun and colleagues showed that it promotes SG assembly in OGD/R models through a mechanism involving TIA-1 and G3BP1, reducing neuronal death in a condensate-dependent manner (Sun et al., 2024). This is a phenotypic association between enhanced SG assembly and reduced injury. It is not the kind of specificity demonstration described above for caspase sequestration. TIA-1 and G3BP1 each have translational and signaling roles outside SGs. A SG-localization-deficient control comparable to the mutant caspase-3 construct used by Fujikawa and colleagues has not yet been reported for muscone. Remote ischemic conditioning is a non-pharmacological approach in which brief cycles of limb ischemia and reperfusion are applied to a remote limb. When delivered early after stroke onset, it was associated by Wang and colleagues with preserved SG integrity in neurons and attenuated NLRP3 activation (Wang et al., 2024). This suggests that early conditioning may act partly by reinforcing the protective SG response rather than solely through hemodynamic or humoral routes. Because conditioning is a systemic physiological intervention rather than a molecularly targeted one, its SG-specific contribution cannot be isolated with the tools reported so far. Icariin is a flavonoid glycoside with established anti-ischemic properties. Li and colleagues showed that it binds G3BP1 and shifts its condensate topology toward an assembly-competent state (Li et al., 2026). This promotes SG formation and recruits the m6A reader IGF2BP1. The resulting hub raises GPX4 protein through an m6A-dependent axis, which couples protection against apoptotic and ferroptotic death within one molecular framework. This causal claim rests on the G3BP1 and IGF2BP1 knockdown experiments described above. Those experiments abolished the GPX4 increase and tie the effect to the SG route rather than to an independent action of icariin. Among the strategies characterized so far, icariin is the most mechanistically integrated. Its effects converge on the G3BP1 scaffold itself rather than on upstream signaling whose specificity for the SG response is less certain.

### 3.10 Disassembly and resolution of pathological condensates in the subacute phase

The therapeutic objective shifts once solidification has begun. In the subacute phase, the priority is no longer to reinforce assembly but to restore the fluidity of aging granules or to accelerate their clearance before irreversible aggregation sets in. The evidence here is more limited, yet it identifies several targets of potential value.

Melatonin was reported by Lu and colleagues to dissolve refractory solid-phase SGs in brain vascular endothelial cells under prolonged ischemia-reperfusion, restoring function in a manner consistent with reactivated disassembly machinery (Lu et al., 2023). Whether this effect extends to neurons, and whether it acts through a direct physical mechanism or through induced chaperone expression, remains to be determined. The upregulation of HSP70 represents a second strategy. Substantial evidence from ALS and frontotemporal dementia research shows that HSP70 overexpression suppresses TDP-43 and FUS solidification by maintaining the solubility of their low-complexity domains. Gu and colleagues showed directly that Hsp70 chaperones TDP-43 in a dynamic, liquid-like phase and prevents its amyloid aggregation (Gu et al., 2021). Translation to CIRI is mechanistically plausible given the shared substrates, but direct *in vivo* evidence in ischemic brain has not yet been reported. HDAC6 inhibition presents a further candidate, although its rationale rests on broader cytoprotective effects rather than enhanced aggregate clearance. Selective HDAC6 inhibitors such as tubastatin A reduce infarct volume and improve functional outcomes in rodent MCAO models, an effect attributed mainly to restored α-tubulin acetylation and microtubule-based transport (Wang et al., 2016). A systematic review of 71 animal studies across HDAC and sirtuin modulators found non-selective HDAC inhibition and HDAC6 inhibition specifically among the more promising approaches for reducing infarct volume (Majdi et al., 2026). Additional work links HDAC6 interference to anti-oxidative signaling through the Nrf2 and HO-1 pathway (Li et al., 2019). Positron emission tomography imaging shows HDAC6 expression declining on the first day after ischemia before rising over the following week. A novel brain-permeable inhibitor administered in this window reduced infarct size and neuroinflammation in mice (Zhou et al., 2024). This rationale requires caution. HDAC6 is itself required for aggresome formation and for the autophagic clearance of ubiquitinated aggregates. Its inhibition in CIRI therefore reflects a context-dependent balance between the loss of this clearance function and the gain of improved transport and redox protection.

### 3.11 Emerging strategies targeting phase transition regulators

Beyond the compounds above, several conceptually distinct strategies deserve attention. The work of Li and colleagues on icariin introduced the notion that the condensate topology of G3BP1 can be pharmacologically modulated (Li et al., 2026). Topology here means the spatial organization of the multivalent interaction network within the granule rather than simply the expression level. This concept opens the possibility of designing small molecules that tune the physical properties of the SG scaffold without abolishing its assembly capacity (Deka et al., 2026). Such an approach might preserve the protective functions of SGs while reducing their propensity for pathological solidification. The view of condensates as a nexus of cellular stress, protein aggregation disease, and aging supports this aim and frames the broader rationale for targeting phase behavior (Alberti and Hyman, 2021; Lian and Nan, 2026). Nanocarrier-based delivery of molecular chaperones or their peptide mimetics into the ischemic territory represents a complementary avenue. Its theoretical advantage is concentrating the intervention where condensate aging occurs, though meaningful blood-brain barrier penetration under the altered permeability of reperfusion remains a practical obstacle.

### 3.12 Limitations and translational barriers

An honest assessment reveals limitations substantial enough to preclude any premature claims about clinical readiness. Every pharmacological finding discussed here derives from cell culture models of OGD or from rodent MCAO. None has been evaluated in primates or human tissue. The gap between rodent and human SG biology is not trivial. Differences in the expression and domain architecture of proteins such as TDP-43 and FUS, and species differences in the chaperone repertoire, could alter the dose-response relationships and time dependencies seen in preclinical work.

A second limitation concerns the temporal conflict inherent in a two-phase strategy. The acute and subacute interventions pursue opposing objectives, the first to stabilize assembly and the second to dissolve condensates. Their sequential deployment depends on defining the transition between the protective and pathological phases with sufficient precision. As noted earlier, this critical window has not been defined in human CIRI. This makes the rational design of a staged intervention premature without additional clinical and biomarker data.

A third concern relates to the multifunctional nature of the targets. G3BP1, TDP-43, FUS, and DDX3X each perform essential roles in RNA metabolism beyond SG dynamics. Interventions that broadly alter their behavior risk disrupting functions needed for neuronal homeostasis under non-stressed conditions. Developing strategies with sufficient selectivity for condensate-associated vs. non-condensate-associated pools remains an unresolved technical challenge. Finally, most available data come from male rodents at a single age point. The extent to which age-related changes in chaperone capacity, baseline proteostatic burden, and hormonal environment alter the SG response to CIRI has not been adequately addressed.

## 4 Conclusion and future perspectives

The evidence reviewed here supports a reframing of stress granules in CIRI. SGs are not incidental markers of cellular stress. They act as dynamic checkpoints at which neurons integrate translational, proteostatic, and signaling information during the acute and subacute phases of injury. The physical state of the condensate is an active determinant of neuronal fate rather than a passive reflection of injury severity. A fluid condensate supports cytoprotective sequestration and regulated disassembly. An irreversible solidification, driven by oxidative modification and chaperone insufficiency, commits the neuron to proteostasis collapse. The three protective functions identified within liquid-state SGs operate upstream of the individual death programs that have dominated neuroprotection efforts. The first is sequestration of executioner caspase-3 and caspase-7, a mechanism identified in cancer-cell and xenograft models rather than in CIRI itself. The second is upregulation of GPX4 protein through a G3BP1-IGF2BP1-m6A hub, demonstrated directly in ischemic neurons with causal knockdown validation. The third is reduction of DDX3X availability for NLRP3 inflammasome assembly, now supported by direct biochemical evidence in ischemic brain tissue rather than by extrapolation from macrophages alone. This upstream position is what makes the SG checkpoint mechanistically and therapeutically relevant. It offers a point at which multiple downstream cascades might be addressed through a single strategy.

Three questions stand out for the field. The first is the time window for intervention in human CIRI. Animal data place it broadly in early reperfusion but cannot yet define it, which calls for non-invasive biomarkers of solidification state. The second is cell-type selectivity. Endothelial and astrocytic solidification are now documented independently of the neuronal data, but no study has yet compared solidification kinetics across these compartments within the same CIRI model. Single-cell studies of SG composition and phase behavior across the neurovascular unit could close this gap. The third is whether condensate topology targeting, exemplified by icariin, can be advanced into a viable compound series through primate validation and structural characterization of the G3BP1 interface. As proximity ligation assays, cryo-electron tomography, and single-molecule imaging become more accessible in disease-relevant models, these questions move within reach. This positions the SG checkpoint as a productive focus for the next stage of CIRI neuroprotection research.
